# Effects of Ramadan Intermittent Fasting on the Severity of β-Thalassemia Major Patients

**DOI:** 10.7759/cureus.36735

**Published:** 2023-03-27

**Authors:** Anas Ibraheem

**Affiliations:** 1 Internal Medicine, Imamein Kadhimein Medical City, Baghdad, IRQ

**Keywords:** medicine, hereditary disorder, baghdad, intermittent fasting, packed red blood cell transfusion, thalassemia & hemoglobinopathies, liver function, clinical hematology, ramadan fasting, β-thalassemia major

## Abstract

Background: β-thalassemia major (β-TM) is an inherited autosomal recessive disorder manifested by the hemoglobin β chain synthesis alteration. It is a lifelong illness with a scope of a wide range of complications. Many kinds of literature evaluated the effect of Ramadan intermittent fasting (RIF) on different medical conditions. However, there are no precise guidelines regarding its effect on patients with β-TM.

Methods: A retrospective cohort study was conducted on β-TM patients who visited the hereditary blood disorder center at Al Karama Teaching Hospital in Baghdad. Accordingly, six parameters were used to evaluate the effect of RIF on β-TM patients before, during, and after Ramadan. These parameters include hemoglobin level, frequency of transfusion, aspartate aminotransferase (AST) level, alanine aminotransferase (ALT) level, left ventricular ejection fraction % (EF%), and spleen size. All of these details, including the demographic characteristics of age, gender, history of splenectomy, and body mass index (BMI) were retrieved from the patient’s medical records after confirming their fasting through one-to-one interviews. This study aimed to fill the gap and investigate the possible effect of RIF on the severity of β-TM.

Results: A total of 184 β-TM patients were enrolled in this study. The mean duration of fasting was 25.2±2.18 days. More than half (110) of the patients were females (59.8%). Whereas, the mean age was 24.8±3.5 years. One-third of the patients (65) had a splenectomy (35.3%) and more than two-thirds had normal BMI. The initial parameters used to score the severity of β-TM were evaluated separately. As a result, the hemoglobin level remained steady without any statistical significance during the three months. In addition, the frequency of blood transfusion and the spleen size carried the same result. Although the lowest median and range of liver enzymes were recorded during Ramadan, they were statistically insignificant compared to pre and post-fasting. Moreover, the left ventricular EF% was insignificant regardless of the patient's history of heart disease.

Conclusion: This study revealed that RIF does not seem to affect the severity of β-TM if the patients proceed with fasting. However, further studies in more countries with a bigger sample size are recommended to confirm these findings.

## Introduction

β-thalassemia major (β-TM) is an inherited autosomal recessive disorder caused by either reduction or absence in β-globin chain synthesis [1]. Despite the rare dominant forms, patients with β-TM are either homozygotes or compound heterozygotes for β0 or β+ genes [2,3]. The imbalance in the β-globin chain ratio results in ineffective erythropoiesis, chronic hemolytic anemia, compensatory hemopoietic expansion, hypercoagulability, and increased intestinal iron absorption [4]. Individuals with β-TM, the most severe type of thalassemia, are usually diagnosed during the first two years of life when they present with microcytic anemia, mild jaundice, and hepatosplenomegaly [5-7].

Thalassemia can be diagnosed by various laboratory investigations including genetic analysis, full blood count, blood film, iron studies, prenatal testing, and hemoglobin electrophoresis [8]. The current management for the vast majority of β-TM patients would be regular blood transfusions along with effective chelating therapy. Nevertheless, other therapy methods are available including splenectomy, bone marrow and hematopoietic stem cell transplantation, gene therapy, and induction of fetal hemoglobin production [9,10].

β-TM is a lifelong illness with a wide range of complications that are either related to frequent blood transfusion or the disease per se. A few of these complications are transfusion-transmitted infections, growth retardation, iron overload, osteoporosis, and dysfunction of the heart and the endocrine system among others [11,12]. The prevalence of β-TM is the highest among Mediterranean countries such as the Middle East, Central Asia, and Southern China. Consequently, Islamic countries have a high prevalence of the disease [7,13-16].

In Islam, fasting during Ramadan is one of the five essential pillars. Muslims abstain from ingesting food and liquids from dawn to sunset for a month-long period. In a typical fast, Muslims have a pre-dawn meal and another post-sunset to break their fast [17-19]. In many kinds of literature, the effect of Ramadan intermittent fasting (RIF) on different medical conditions was evaluated [20-23]. However, there are no precise guidelines that would help physicians decide whether to advise patients with β-TM to practice fasting during Ramadan or not.

Although Islam exempts sick people from fasting, many Muslims would choose to partake in this religious month. Therefore, the purpose of this study was to fill the gap and investigate the possible effect of RIF on the severity of β-TM. Assessing the severity of thalassemia, independently by genotype and globin chain imbalance, is not clear except for a few attempts to settle for a severity scoring system in two published literature [24,25]. Accordingly, a few adjustments were made by the author to the available two scoring systems in order to ensure the final parameters would serve the aim of this study. The final scoring system was composed of six parameters which are interpreted and discussed elaborately in the methodology afterward.

## Materials and methods

Study design and settings

A retrospective cohort study was conducted on β-TM patients who visited the hereditary blood disorder center at Al Karama Teaching Hospital in Baghdad, Iraq. This study included all patients aged ≥18 years who fasted more than 20 days of Ramadan (corresponding to the Gregorian date from 2 April to 1 May 2022). This timeline was chosen to exclude the predictable days of blood transfusion and females' menses as a result of Islam's exemption from fasting during such situations. Moreover, the fasting duration each day was approximately 14 hours. It is worth mentioning that patients who were pregnant or positive for COVID-19, HIV, hepatitis C virus (HCV), and hepatitis B virus (HBV) were excluded from this study. The participants’ diagnoses of β-TM were confirmed based on clinical and genetic testing.

Methods and data collection

The effect of RIF was assessed according to the parameters of the two available published β-TM severity scoring systems [24,25]. A few adjustments were edited by the author in order to ensure the final parameters would serve the aim of this study. Therefore, these parameters (hemoglobin level, frequency of transfusion, aspartate aminotransferase (AST) level, alanine aminotransferase (ALT) level, left ventricular ejection fraction % (EF%), and spleen size) were studied one month before, during, and after Ramadan. All of these details, including the demographic characteristics of age, gender, history of splenectomy, and body mass index (BMI), were retrieved from the patient’s medical records after confirming their fasting through one-to-one interviews. In addition, verbal consent was obtained along with a pledge only to use their information for research and scientific purposes. Consequently, 184 β-TM patients of a convenient sample were enrolled in this study.

The parameters, mentioned earlier, were measured at the end of each month. BMI was calculated according to the following formula: BMI=weight (kg)/height (m)^2^. The World Health Organization criteria were adopted to classify their BMI into categories [26]. Whereas, the spleen size was assessed by ultrasonography by measuring the longitudinal spleen length distally from the left costal margin. Moreover, the hemoglobin levels were taken before the blood transfusion sessions.

Statistical analysis

The collected data were analyzed using Statistical Product and Service Solutions (SPSS) (IBM SPSS Statistics for Windows, Version 26.0, Armonk, NY). They were represented in different ways of measurement including frequency, percentage, median, mean, and standard deviation (SD). On the other hand, the statistical significance was determined at p-value < 0.05 using repeated measures of one-way analysis of variance (ANOVA).

## Results

This study included 184 β-TM patients who visited the hereditary blood disorder center at Al Karama Teaching Hospital for three months, from the beginning of March to the end of May 2022. More than half (110) of patients were females (59.8%). Whereas, the mean age was 24.8±3.5 years with a median of 25 (range 18-43). On the other hand, the mean duration of fasting was 25.2±2.18 days with a median of 25.5 (range 21-28). Furthermore, one-third of patients (65) had a splenectomy (35.3%), and more than two-thirds had normal BMI (for details, see Table 1).

**Table 1:** General profile and characteristics of the enrolled β-TM patients (n=184). β-TM: β-thalassemia major; BMI: body mass index; SD: standard deviation

Variable		Value
Gender	Male	74 (40.2%)
	Female	110 (59.8%)
Age (years)	Mean±SD	24.8±3.5
	Median (range)	25 (18-43)
Fasting duration (days)	Mean±SD	25.2±2.18
	Median (range)	25.5 (21-28)
Splenectomy	Yes	65 (35.3%)
	No	119 (64.7%)
BMI (kg/m^2^)	Underweight	43 (23.3%)
	Normal weight	132 (71.7%)
	Overweight	7 (4%)
	Obese	2 (1%)
	Mean±SD	20.4±2.17

The initial parameters used to score the severity of β-TM were evaluated separately before, during, and after Ramadan. As a result, the hemoglobin level remained steady without any statistical significance. Similarly, the frequency of blood transfusion during each month was statistically insignificant with a median of three times. The spleen size was assessed by ultrasonography for 119 patients. Consequently, there was no significant difference in the size during the three months.

Regardless of whether the patient had liver disease or not, no significant change was found in the levels of liver enzymes. However, the lowest median and range of AST and ALT were recorded during Ramadan at 68.5 U/L (40.2-147) and 77.8 U/L (56-162), respectively. On the other hand, the left ventricular EF% was insignificant regardless of the patient's history of heart disease (for details, see Table 2).

**Table 2:** Effect of Ramadan intermittent fasting on the severity of β-TM patients. β-TM: β-thalassemia major; RIF: Ramadan intermittent fasting; AST: aspartate aminotransferase; ALT: alanine aminotransferase; EF: ejection fraction; SD: standard deviation

Variable		Before RIF	During RIF	After RIF	P-value
Hemoglobin level (g/dL)	Mean±SD	8.2±0.88	8±0.89	8.1±0.97	0.44
	Median (range)	8.3 (6.7-11.5)	7.9 (6.4-10.5)	7.8 (6.5-10.2)	
Frequency of blood transfusion (times/month)	Mean±SD	2.63±0.87	2.75±0.83	2.68±0.86	0.71
	Median (range)	3 (1-4)	3 (1-4)	3 (1-4)	
Spleen size (cm)	Mean±SD	9.2±5.72	9.5±5.74	10.3±5.76	0.59
	Median (range)	8.4 (2-21)	9 (2-21.3)	9.3 (3-20.8)	
AST (U/L)	Mean±SD	82.2±25.4	79.8±23.2	78.4±21.1	0.19
	Median (range)	75.8 (48-151)	68.5 (40.2-147)	70.4 (46-148)	
ALT (U/L)	Mean±SD	79.3±20.2	80.8±19.9	82.5±18.2	0.23
	Median (range)	80.3 (61-192)	77.8 (56-162)	84 (67-195)	
EF (%)	Mean±SD	52.3±10.5	53.9±9.6	50.6±7.2	0.85
	Median (range)	54 (42-65)	56 (45-63)	52 (41-60)	

## Discussion

In Ramadan, Muslims fast during daytime hours by restriction of food and drink intake along with changes in the circadian distribution of sleeping and eating schedules. This would result in numerous alterations in the metabolism of the human body [27]. Although several kinds of literature discussed the effect of RIF on various medical conditions, there was no well-recognized published data regarding its influence on β-TM patients.

In this study, the hemoglobin levels were found to be steady and without statistical significance (p-value=0.44) when comparing records before, during, and after Ramadan. Similar to two trials conducted on healthy subjects in Jordan and Iran [28,29]. Furthermore, another two studies conducted on patients with sickle cell anemia and chronic myeloid leukemia revealed the same conclusion [30,31]. However, unlike a study conducted in Saudi Arabia where the hemoglobin level was higher at the end of Ramadan than after a month [32]. On the other hand, RIF was significantly associated with decreased hemoglobin levels when compared to either after-Ramadan records in a study in Tunisia or before Ramadan in a study conducted in Iran [33,34]. This discrepancy in the hemoglobin levels might be attributed to the different study samples and the medical backgrounds of each study. In addition, the variation in the economic, geographical, climatic, and nutritional factors should be considered.

According to the results of the present study, The frequency of blood transfusion was statistically insignificant and remained the same with a median of three transfusion sessions during each month. This result can be related to the idea that roughly the frequency of blood transfusion disproportionally correlates to the hemoglobin level. Therefore, steady levels of hemoglobin made a regular frequency of transfusion more likely [35,36]. However, other factors that determine the rate of transfusion in β-TM patients had not been discussed as a change in this study which may reflect a limitation to this assumption. These factors include facial changes, growth retardation, spontaneous fractures, and clinically significant extramedullary hematopoiesis [37].

In this study, RIF had no effect on the spleen size. Some evidence of lifestyle interventions including dietary modifications can reduce the enlarged spleen, but no human studies have directly understood how the diet would affect spleen size [38]. Although the liver enzymes in our results had no statistical significance, the lowest AST and ALT levels were recorded during Ramadan at 68.5 U/L (40.2-147) and 77.8 U/L (56-162), respectively. This finding is consistent with a study conducted in Iran [39]. Conversely, ALT levels increased during RIF in another study conducted on patients with nonalcoholic fatty liver disease (NAFLD) [40]. A systematic review with meta-analysis of different six studies showed the beneficial effects of fasting on liver enzymes suggesting to be related to the reduction in body weight and/or body fat [41]. Interestingly, other trials have shown the same conclusion [42-44]. This discrepancy from our study might be attributed to the fact that β-TM patients had already low-fat storage and low body mass index prior to fasting. Moreover, both direct and indirect effects of the disease per se including iron overload might affect the liver response to fasting compared to the subjects in the aforementioned studies.

Finally, the left ventricular EF% remained within the same percentage among the patients in question (p-value=0.85). It is consistent with previous studies for both heart failure with reduced and preserved EF published in 2018 and 2022, respectively [45,46]. These findings might be assigned to the effect of Intermittent fasting on increasing galectin-3, a key protein that controls inflammation and protects the heart, according to a primary and secondary analysis of two randomized control trials [47,48]. It is the same protein that was found to be raised when patients were commenced on sodium-glucose co-transporter 2 (SGLT-2) inhibitors [47]. The study's lead researcher, Dr. Benjamin Horne, hypothesized the process of intermittent fasting might be similar to SGLT-2 inhibitors in lowing the risk factors for heart failure and type 2 diabetes [49]. However, these trials adopt a different definition of intermittent fasting, which may last for up to 26 weeks. Therefore, the acceptance of this assumption per se in our study might be limited.

## Conclusions

This study revealed that RIF does not seem to affect the severity of β-TM if the patients proceed with fasting. Nevertheless, the limitations of this study being conducted retrospectively in a limited geographical region with a convenient patient sampling might restrict the generalization of these findings and raise the unintentional risk of bias. Therefore, randomized control trials in more countries with a bigger sample size are recommended to overcome these obstacles in future studies.
